# Skin delivery of epigallocatechin-3-gallate (EGCG) and hyaluronic acid loaded nano-transfersomes for antioxidant and anti-aging effects in UV radiation induced skin damage

**DOI:** 10.1080/10717544.2016.1228718

**Published:** 2017-02-03

**Authors:** Kiran S. Avadhani, Jyothsna Manikkath, Mradul Tiwari, Misra Chandrasekhar, Ashok Godavarthi, Shimoga M. Vidya, Raghu C. Hariharapura, Guruprasad Kalthur, Nayanabhirama Udupa, Srinivas Mutalik

**Affiliations:** 1 Department of Pharmaceutics, Manipal College of Pharmaceutical Sciences, Manipal University, Manipal, India,; 2 Department of Pharmaceutical Biotechnology, Manipal College of Pharmaceutical Sciences, Manipal University, Manipal, India,; 3 Radiant Research Services Pvt. Ltd, Peenya Industrial Area, Bangalore, India,; 4 Department of Biotechnology, NMAM Institute of Technology, Nitte University, Nitte, India, and; 5 Department of Clinical Embryology, Kasturba Medical College, Manipal University, Manipal, India

**Keywords:** Epigallocatechin-3-gallate (EGCG), hyaluronic acid, transfersomes, polyphenols, antioxidant, skin permeation

## Abstract

The present work attempts to develop and statistically optimize transfersomes containing EGCG and hyaluronic acid to synergize the UV radiation-protective ability of both compounds, along with imparting antioxidant and anti-aging effects. Transfersomes were prepared by thin film hydration technique, using soy phosphatidylcholine and sodium cholate, combined with high-pressure homogenization. They were characterized with respect to size, polydispersity index, zeta potential, morphology, entrapment efficiency, Fourier Transform Infrared Spectroscopy (FTIR), Differential Scanning Calorimetry (DSC), X-ray Diffraction (XRD), *in vitro* antioxidant activity and *ex vivo* skin permeation studies. Cell viability, lipid peroxidation, intracellular ROS levels and expression of MMPs (2 and 9) were determined in human keratinocyte cell lines (HaCaT). The composition of the transfersomes was statistically optimized by Design of Experiments using Box–Behnken design with four factors at three levels. The optimized transfersome formulation showed vesicle size, polydispersity index and zeta potential of 101.2 ± 6.0 nm, 0.245 ± 0.069 and −44.8 ± 5.24 mV, respectively. FTIR and DSC showed no interaction between EGCG and the selected excipients. XRD results revealed no form conversion of EGCG in its transfersomal form. The optimized transfersomes were found to increase the cell viability and reduce the lipid peroxidation, intracellular ROS and expression of MMPs in HaCaT cells. The optimized transfersomal formulation of EGCG and HA exhibited considerably higher skin permeation and deposition of EGCG than that observed with plain EGCG. The results underline the potential application of the developed transfersomes in sunscreen cream/lotions for improvement of UV radiation-protection along with deriving antioxidant and anti-aging effects.

## Introduction

Exposure to ultra-violet radiations (UVR) has been implicated in pathological conditions like skin cancer and premature skin aging. UV radiation in the wavelength ranges of 280–320 nm (UVB) and 320–400 nm (UVA) causes premature photoaging, immune suppression (by alterations in IL-10 and IL-12 production) and skin carcinogenesis (by mutation of p53 suppressor gene and activation of reactive oxygen species (ROS), resulting in DNA damage (Katiyar & Mukhtar, 2001; Vayalil et al., 2003; Beissert & Loser, 2008). UV radiations also damage other critical macromolecules like proteins and lipids in the skin. Broad spectrum physical and chemical sunscreens such as titanium dioxide, zinc oxide, octylmethoxycinnamate, oxybenzone, octocrylene, aminobenzoic acid, avobenzone, cinoxate, dioxybenzone, homosalate and menthyl anthranilate have been widely used to prevent UVR exposure. However these physical and chemical sunscreens have been reported to cause skin irritation, photosensitivity and contact dermatitis by interaction with cutaneous molecules upon chronic use (Nohynek & Schaefer, 2001). Therefore, as a result of their better tolerability and lesser impact on the environment, greater impetus is being given to natural compounds like polyphenols for their photoprotective properties (Gaspar & Maia Campos, 2006; Gaspar & Campos, 2007; Beissert & Loser, 2008).

Epigallocatechin-3-gallate (EGCG) is an effective antioxidant, most abundantly available in green tea. EGCG has captured a lot of research interest due to its wide range of biological effects like antioxidant, photo protective, anti-aging, anti-inflammatory, immune modulatory, anticancer, neuroprotective, cardioprotective, antiviral and antibacterial effects (Fang et al., 2005; Brand & Jendrzejewski, 2008). Many of these properties make EGCG, a potential candidate in sunscreen formulations and several studies bear testimony to this fact (Zhu et al., 2014). However, its clinical use has been limited because of its poor systemic absorption, resultant low bioavailability and strong systemic clearance upon oral administration. Therefore in this study, we developed nano-transfersomal formulations of EGCG, for their efficient permeation into the stratum corneum and effective delivery of EGCG into the skin (Chow et al., 2005).

Along with EGCG, we sought to encapsulate another bioactive molecule, hyaluronic acid (HA) in the nano-transfersomes. HA is widely distributed in connective tissues and is the main component of extracellular matrix (Prikhnenko, 2015). It is a nonirritating biopolymer that has been reported as an effective anti-aging agent because of its properties like wound healing, skin repair, skin hydration and protection against skin wrinkling. It is also used in the treatment of skin ulcers. Specific properties of HA, including biocompatibility, specific viscoelasticity, hydration and lubrication, make it a widely used polymer in the pharmaceutical and cosmetic industries (Atrux-Tallau et al., 2014).

Lipid based nano-carriers such as liposomes, nanoemulsions, microemulsions, ethosomes, transfersomes and solid lipid nanoparticles have been widely reported for skin delivery of bioactive molecules (Atrux-Tallau et al., 2014). Transfersomes or deformable liposomes are one of the suitable nano-carriers for skin delivery of drugs. They are modified liposomes prepared with phospholipids and edge activators (such as sodium cholate (SC) and sodium deoxycholate), which can efficiently increase the skin permeation of drugs (Cevc & Blume, 2001).

Thus, the present study describes the co-encapsulation of EGCG and HA in a single nano-transfersomal drug carrier system for obtaining synergistic antioxidant and anti-aging benefits of both compounds. The transfersome formulations were optimized by Design of Experiments (DoE) using Box–Behnken design and evaluated with respect to various parameters including skin retention and permeation.

## Materials and methods

### Materials

Epigallocatechin-3-gallate, phospholipon 90G (high purity soy phosphatidyl choline, SPC), 1,1-diphenyl-2-picrylhydrazyl radical, 2,2-diphenyl-1-(2,4,6-trinitrophenyl) hydrazyl (DPPH), 2,2′-azino-bis(3-ethylbenzothiazoline-6-sulphonic acid) (ABTS), MTT (3-(4,5-dimethylthiazol-2-yl)-2,5-diphenyltetrazolium bromide), HA, 2′,7′-dichlorofluorescein diacetate (H_2_DCFDA) and SC were purchased from Sigma Aldrich, St. Louis, MO. Chloroform, methanol and acetonitrile (HPLC grade) were purchased from Merck Chemicals, Mumbai, India. All other chemicals used were of analytical/reagent grade.

#### Cell lines and culture media

HaCaT (human keratinocytes) cells were procured from National Centre for Cell Sciences (Pune, India). The cells were cultured with Dulbecco’s Modified Eagle’s Medium supplemented with 10% fetal bovine serum, penicillin (100 μg/mL), streptomycin (100 μg/mL) and amphotericin-B (5 μg/mL), and incubated in 5% CO_2_ at 37 °C (Healforce Incubator, Shanghai, China). The cells were dissociated with Trypsin Phosphate Versene Glucose (TPVG) solution (0.2% trypsin, 0.02% EDTA, 0.05% glucose in phosphate buffered saline). The stock cultures were grown in 25 cm^2^ culture flasks and all the experiments were carried out in 60 mm petriplates (Tarsons India Pvt. Ltd., Kolkata, India).

#### Animals

Preclinical experimental protocol was approved by Institutional Animal Ethical Committee, Manipal University, Manipal (IAEC/KMC/101/2012). Male Wistar rats, weighing 200 ± 20 g, were supplied from Central Animal House Facility, Manipal University, Manipal, India. The animals were maintained in separate cages (four per cage) at 25 °C with 12/12 h light/dark cycle. Food and water were provided *ad libitum*. To reduce the stress associated with the experimental procedure, the rats were handled daily for one week prior to experimentation.

### Methods

#### Preparation and optimization of transfersomes

Transfersomes were prepared by modified thin film hydration method followed by high-pressure homogenization technique (Rattanapak et al., 2012). SPC, EGCG and SC were dissolved in the mixture of chloroform:methanol (4:1 v/v) in a round bottomed flask. The solvent was removed under reduced pressure using Rotavap (Buchi, Switzerland) until a lipid film was formed on the inner-wall of the flask. Hydration of the lipid film was achieved by adding 10 mL of phosphate buffer pH 6.8 containing HA. After the film was completely removed, the mixture was passed through a High-Pressure Homogenizer (EmulsiFlex-C3, Avestin, Canada) at a pressure of 1000–1200 bars for 10 cycles. The vesicles were collected by centrifugation at 22 000 rpm for 45 min at 4 °C. The pellet obtained was dispersed in Milli-Q water. The formulations were optimized by applying DoE approach (Ahad et al., 2012). Box–Behnken design with four factors at three levels was selected to execute the quadratic response surfaces to construct second order polynomial models using Design Expert software (Version 9.0.3.1; Stat Ease, Minneapolis, MN) and accordingly, 29 trials were planned (Table 1). Apart from these 29 formulations, one more formulation was planned with EGCG alone (EGCG-TF). The composition of this formulation was the same as that of ETF20 (an optimized formulation) with the exclusion of HA. The independent variables selected in this study were: i) amount of phospholipids (*X*_1_), ii) amount of surfactant (*X*_2_), iii) amount of EGCG (*X*_3_) and iv) amount of HA (*X*_4_). The dependent variables were: i) vesicle size (*Y_1_*), ii) polydispersity index (*Y_2_*), iii) zeta potential (*Y_3_*), iv) % Entrapment Efficiency (EE) of EGCG (*Y_4_*) and v) % EE of HA (*Y_5_*). Polynomial equations were generated for all the dependent variables using multiple linear regression analysis (Mahmood et al., 2014).

**Table 1.** Composition of transfersomal formulations and observed responses in Box–Behnken design for transfersomal formulations containing EGCG and HA.

Batches	SPC (mg)	SC (mg)	EGCG (mg)	HA (mg)	Size^a^ (nm)	Zeta potential^a^ (mV)	EE of EGCG^a^ (%)	EE of HA^b^ (%)
ETF1	85	5	5	1.75	101.7 ± 4.16	−60.7 ± 3.04	66.31 ± 1.76	30.68 ± 3.04
ETF2	85	15	15	3	176.7 ± 8.20	−52.9 ± 4.14	64.10 ± 1.43	35.70 ± 3.85
ETF3	95	15	10	0.5	91.7 ± 5.13	−36.3 ± 1.14	64.69 ± 2.98	15.00 ± 1.56
ETF4	85	25	10	3	105.7 ± 6.29	−46.5 ± 2.94	49.36 ± 2.60	21.85 ± 2.14
ETF5	85	15	5	0.5	103.7 ± 7.53	−52.0 ± 3.87	57.63 ± 2.24	12.00 ± 1.30
ETF6	85	25	15	1.75	206.8 ± 12.03	−48.8 ± 3.56	54.89 ± 1.78	21.60 ± 1.90
ETF7	85	15	10	1.75	105.7 ± 4.40	−43.5 ± 5.32	73.08 ± 2.34	43.14 ± 4.23
ETF8	95	25	10	1.75	122.7 ± 7.16	−45.7 ± 4.59	46.84 ± 1.56	18.45 ± 1.34
ETF9	75	15	10	0.5	131.3 ± 6.38	−44.1 ± 4.32	73.57 ± 1.67	57.00 ± 4.98
ETF10	75	25	10	1.75	109.6 ± 5.27	−53.3 ± 4.34	74.42 ± 2.45	36.57 ± 2.15
ETF11	95	5	10	1.75	112.8 ± 6.42	−45.1 ± 4.35	51.88 ± 1.15	17.48 ± 2.19
ETF12	85	5	10	0.5	112.1 ± 5.50	−41.2 ± 3.89	62.21 ± 4.40	19.56 ± 2.20
ETF13	95	15	15	1.75	234.6 ± 8.71	−45.1 ± 4.10	43.30 ± 2.76	21.70 ± 1.28
ETF14	85	15	10	1.75	107.4 ± 5.98	−47.2 ± 4.45	73.22 ± 2.37	46.20 ± 4.97
ETF15	75	15	10	3	164.0 ± 8.27	−45.6 ± 3.82	46.10 ± 1.16	27.73 ± 3.20
ETF16	75	15	5	1.75	96.8 ± 3.44	−39.9 ± 3.75	72.63 ± 2.89	32.85 ± 3.10
ETF17	85	15	5	3	132.8 ± 5.03	−48.9 ± 4.26	67.36 ± 1.65	23.04 ± 3.56
ETF18	85	5	15	1.75	187.5 ± 9.66	−47.8 ± 4.98	63.20 ± 2.23	27.98 ± 3.56
ETF19	85	15	10	1.75	103.3 ± 5.34	−41.6 ± 4.38	75.00 ± 1.45	46.00 ± 2.60
ETF20	85	15	10	1.75	101.2 ± 4.82	−44.8 ± 4.24	76.53 ± 2.68	48.57 ± 4.35
ETF21	85	15	15	0.5	145.2 ± 8.25	−43.2 ± 3.80	49.50 ± 3.50	19.20 ± 2.39
ETF22	85	25	5	1.75	93.55 ± 4.73	−30.6 ± 3.69	54.73 ± 3.92	53.14 ± 4.78
ETF23	95	15	10	3	120.3 ± 5.92	−52.5 ± 5.23	58.73 ± 4.15	41.35 ± 1.26
ETF24	95	15	5	1.75	91.5 ± 6.12	−42.5 ± 4.20	51.15 ± 1.70	17.14 ± 2.44
ETF25	85	5	10	3	113.5 ± 4.58	−46.1 ± 5.56	56.89 ± 2.80	26.00 ± 1.50
ETF26	75	5	10	1.75	135.8 ± 6.74	−47.1 ± 4.85	42.21 ± 2.34	9.00 ± 1.180
ETF27	75	15	15	1.75	159.2 ± 7.83	−54.1 ± 4.84	59.50 ± 1.50	24.80 ± 3.28
ETF28	85	25	10	0.5	109.5 ± 4.37	−58.1 ± 4.98	47.36 ± 3.60	25.00 ± 2.27
ETF29	85	15	10	1.75	108.5 ± 3.61	−46.1 ± 4.15	73.89 ± 1.58	41.64 ± 1.58
EGCG-TF	85	15	10	–	96.5 ± 5.58	−41.4 ± 3.56	70.58 ± 1.63	–

SPC: soy phosphatidyl choline; SC: sodium cholate; EGCG: epigallocatechin-3-gallate; HA: hyaluronic acid; PDI: polydispersity index; EE: entrapment efficiency.

Values are expressed as mean ± SD; *n* = 3.

^a^ Significantly influenced by independent variables (SPC, SC and EGCG) (*p *< 0.05).

^b^ Not significantly influenced by independent variables (SPC, SC, EGCG and HA) (*p *> 0.05).

#### Analysis of EGCG

HPLC system (LC-2010, Shimadzu, Kyoto, Japan), comprising vacuum degasser, quaternary pump, auto-sampler, thermostatic column compartment, UV-Visible detector and LC Solution software (version 1.24 SP1) was used to analyze EGCG. Reversed-phase chromatography was performed on a C_18_ column (250 mm, 4.6 μm, 5 μm; Phenomenex, Torrance, CA). The mobile phase used was a mixture (25:75 v/v) of acetonitrile and 1% v/v acetic acid in water with pH adjusted to 2.8 using triethylamine. Drug elution was performed at 25 °C with a flow rate of 0.8 mL/min. The injection volume was 20 μL and detection wavelength was 280 nm. The retention time of EGCG was found to be 3.4 min. The developed method was found to be linear (*R*^2^: 0.9998), precise (% RSD < 2%) and accurate (% RSD < 2%). The limit of detection and limit of quantification were found to be 0.125 μg/mL and 0.272 μg/mL, respectively.

#### Characterization of transfersomes

*Vesicle size, zeta potential and morphology*. Size of the transfersomes was determined by dynamic light scattering (DLS) method using Zeta Sizer (NanoZS, Malvern Instruments, Malvern, UK) at 25 ± 0.5 °C. Measurement of the zeta potential of sample in the Nano ZS particle analyser (Malvern Instruments, Malvern, UK) was done using a combination of Laser Doppler Velocimetry (LDV) and Phase Analysis Light Scattering (PALS). The morphology of the optimized formulation was investigated with Transmission Electron Microscopy (TEM; CM200, Philips, Andover, MA) by staining the sample with 2% w/v phosphotungstic acid (adjusted to pH 6.0) for 60 s and then placing the air-dried sample on the copper grid for visualization of vesicles.

*Entrapment efficiency*. A known amount of the vesicles was lysed by 2% v/v Triton X-100 solution. After filtration through 0.22 μm membrane and appropriate dilution, the sample was injected into HPLC system to determine the drug content. Percentage entrapment was calculated by considering the amount of EGCG encapsulated and the total amount of EGCG used.

To determine the content of HA, a known quantity of transfersomal formulation was dissolved in methanol and centrifuged at 10 000 rpm for 10 min. The supernatant was discarded and the residue dissolved in phosphate buffer pH 6.8. The concentration of HA in this solution was estimated in a UV-spectrophotometer (UV-1601PC, Shimadzu, Kyoto, Japan) at 212 nm. The EE of HA was calculated by considering amount of HA encapsulated and total amount of HA used.

*Drug-excipient compatibility studies*. Fourier Transform Infrared Spectroscopy (FTIR), Differential Scanning Calorimetry (DSC) and X-ray Powder Diffraction (XRD) analysis were performed to investigate possible interaction of EGCG and HA with the selected excipients and the crystalline properties (Shetty et al., 2015).

FTIR spectra were recorded using an FTIR spectrophotometer (FTIR-8300, Shimadzu, Japan) at frequencies from 500 to 4000 cm^−1^ to investigate the characteristic bands accompanied with plain EGCG, plain HA, physical mixture of EGCG and HA with excipients and lyophilized transfersome formulation (ETF20).

DSC analysis was performed using DSC-60 (Shimadzu, Japan) instrument. About 4 mg of each sample was sealed in a 40 μL aluminum pan and scanned between 25 and 300 °C, under nitrogen flow (30 mL/min), at a heating rate of 5 mL/min. The samples analyzed were plain EGCG, plain HA, physical mixture of ECGC and HA with excipients and the lyophilized transfersomal formulation (ETF20). DSC thermograms were recorded over the temperature range of 25–300 °C. Sealed empty aluminum pan with lid was used as reference. The above samples were also analyzed by XRD, using an X-ray difractometer (Miniflex-600, Rigaku, Tokyo, Japan) at a scan rate of 2° per minute over the 2*θ* range of 5–60° (Shetty et al., 2015).

#### *In vitro* antioxidant activity

*DPPH assay*: α, α-diphenyl-β-picrylhydrazyl (DPPH) is a stable free radical with strong absorption at 517 nm, scavenged by hydrogen donation from the antioxidant that leads to a decrease in its absorbance. Plain EGCG, transfersomes containing EGCG alone (EGCG-TF) and transfersomes containing EGCG and HA (ETF20) were tested for their antioxidant activity in the concentration range of 0.4–50 μM. Curcumin dissolved in methanol in various concentrations (7.8–250 μM), was used as the standard. The test samples (100 μL) were plated in a 96-transparent well plate. The plate was mixed uniformly and incubated with DPPH in the dark for 20 min at room temperature. Finally, the plate was read at 517 nm using a microplate reader. The assay was performed in triplicate and the inhibitory effect was calculated by using the following formula (Liu et al., 2013).

(1) $$\%\text{Inhibition of DPPH }\;=\;\frac{\text{Absorbance of control }-\text{Absorbance of sample}}{\text{Absorbance of control}}\times 100$$

*ABTS assay.* Free radical scavenging activity was also studied by performing ABTS (2,2′-azino-bis(3-ethylbenzothiazoline-6-sulphonic acid)) assay. ABTS stock solution was prepared by dissolving 2 mM ABTS in water and allowing it to react with 0.17 mM of potassium persulfate in 20 mM of phosphate buffer (pH 7.4). This mixture was allowed to stand in the dark at room temperature for 12–16 h before use. Plain EGCG and transfersomal formulations (EGCG-TF and ETF20) were tested in the concentration range from 0.4 to 50 μM. Curcumin (standard) was solubilized in methanol to get different concentrations (7.8–250 μM). All samples were incubated with ABTS (170 μL) at room temperature for 10 min in the dark. The samples were then read on a microplate reader at 734 nm and the percentage inhibition was calculated (Kumaraswamy & Satish, 2008).

(2) $$\%\text{Inhibition of ABTS }\;=\;\frac{\text{Absorbance of control }-\text{Absorbance of sample}}{\text{Absorbance of control}}\;\times 100$$

#### Cell viability assay

Cell viability study was done by MTT (3-(4,5-dimethylthiazol-2-yl)-2, 5-diphenyltetrazolium bromide) assay. HaCaT cells were plated in a 96-well flat-bottom microtiter plate (density: 1 × 10^4^ cells per well). The cells were cultured for 24 h at 37 °C in 5% CO_2_ to ensure cell adhesion. When partial monolayer was formed (after 24 h), the medium was removed. The cells were exposed to different treatments (increasing concentrations of plain EGCG and EGCG transfersomes (EGCG-TF and ETF20) or control (plain buffer/blank transfersomes)) for 48 h. The cells were observed under microscope after every 24 h. After 48 h of the treatment, the supernatant in the wells was discarded, 50 μL of MTT (2 mg/mL) added to each well and the cells incubated for another 3–4 h. Finally 100 μL of DMSO was added to dissolve the crystals and the plate was read immediately on the plate reader at 540 nm. Experiments were performed in triplicate (Shetty et al., 2015). The % growth inhibition was calculated using the below formula and % cell viability was calculated from the same.

(3) $$\mathrm{Growth}\mathrm{inhibition}\left(\%\right)\;=\;\frac{\text{Control absorbance }-\text{Test absorbance}}{\text{Control absorbance}}\;\times 100$$

#### Lipid peroxidation assay

Lipid peroxidation assay was performed on HaCaT cells in terms of determination of MDA (malondialdehyde), an end product of lipid peroxidation. The cells were seeded in six-well plates at a concentration of 10 000 cells/mL. The treatment groups were:

- Group 1: Control (Untreated cells);
- Group 2: Cells treated with UV irradiation alone (60 J/m^2^ for 5 min using a UV radiation cabinet; Esco, Singapore);
- Group 3: Cells treated with plain EGCG solution (10 μg/mL) along with UV irradiation;
- Group 4: Cells treated with transfersome formulation EGCG TF (containing EGCG alone; 10 μg/mL), along with UV irradiation;
- Group 5: Cells treated with optimized transfersome formulation ETF20 (containing EGCG 10 μg/mL and HA) along with UV irradiation.

After incubation for 48 h, the cells were homogenized and centrifuged at 5000 rpm for 15 min. The absorbance of the supernatant was read spectrophotometrically at 550 nm against blank. Malondialdehyde was determined by using thiobarbituric acid-reactive substance (TBARS) to estimate the extent of lipid peroxidation. The MDA concentration of cells was calculated using an extinction coefﬁcient of 1.56 × 10^5^ M^−1^ cm^−1^. Protein content was determined by BCA assay kit (Vayalil et al., 2003; Liu et al., 2013).

#### Measurement of intracellular ROS level

Intracellular ROS levels in HaCaT cells were determined by measuring the oxidative conversion of cell permeable 2′,7′-dichlorofluorescein diacetate (H_2_DCFDA) to fluorescent dichlorofluorescein (DCF) (Shen et al., 2012). The fluorescence intensity of DCF in HaCaT cells is significantly increased with UVB irradiation, while the background ROS levels in HaCaT cells are low without irradiation. HaCaT cells were cultured in 96-well plates. The treatment groups were same as those mentioned in the lipid peroxidation assay (section "Lipid peroxidation assay").

After incubation for 48 h, the cells were washed with phosphate buffer saline and incubated with 40 μM H_2_DCFDA at 37 °C for 30 min. The cells were then subjected to a multi-functional microplate reader (Molecular Devices, Sunnyvale, CA) for measuring the fluorescence intensity per well at an excitation wavelength of 485 nm and at an emission wavelength of 528 nm (Liu et al., 2013; Zhu et al., 2014).

#### Expression of MMP-2 and MMP-9 by gelatin zymography

The monolayer HaCaT cell culture was trypsinized and the cell count was adjusted to 100 000 cells/mL using DMEM containing 10% FBS. To each petridish, 4 mL of diluted cell suspension was added. After 24 h, when a partial monolayer was formed, the supernatant was removed and the monolayer was washed. Culture medium (4 mL) with 0.5% FBS containing test samples such as retinoic acid (as standard), EGCG-TF and optimized formulation (ETF20) were added onto the partial monolayer in petridish and irradiated with UV-B radiation. Cells treated with UV-B irradiation alone, without drug treatment, served as the positive control. The dishes were then incubated at 37 °C for 24 h in 5% CO_2_ atmosphere, followed by microscopic examination of the cells. Then, the drug solution in the wells was discarded and the treated cells were subjected to isolation of protein. The cells were centrifuged at 10 000 rpm for 15 min and the pellet obtained was dispersed in 0.3 mL of Radio-Immuno-Precipitation Assay **(**RIPA) buffer. The supernatant was retained and concentrated by centrifugation. All the samples were dissolved in the medium and were loaded on Native-PAGE. The samples were kept at room temperature for 5–10 min with sample loading buffer. A short spin allowed the sample to settle easily. SDS-PAGE electrophoresis was carried out using a 10% gel containing 0.1% w/v gelatin. Wells in the stacking gel were washed with distilled water to remove non-polymerized acrylamide. The set was clipped to the electrophoresis apparatus filled with Tris Glycine buffer. The samples were loaded the wells and the apparatus was connected to a power supply. When the dye front came to 0.5 cm above the bottom of the gel, power pack was turned off. The plates were carefully separated apart and the gel was rinsed with distilled water. The gel was transferred to 50 mL of wash buffer and kept in gel rocker for about 30 min. After electrophoresis, the gel was washed with re-naturing buffer (2.5% Triton X-100), kept for 30 min on gel rocker and then equilibrated in developing buffer (50 mM Tris–HCl pH 7.5, 5 mM CaCl_2_, 0.2 M NaCl and 0.2% Brij 35) at room temperature for 30 min. This was again incubated at 37 °C for 24 h in fresh developing buffer for digestion of the gelatin. The gel was washed using distilled water and kept in staining solution (0.5% Coomassie blue G-250 in 5% methanol and 10% acetic acid) for 2 h. The de-stained gel (de-staining solution: 10% methanol, 5% acetic acid v/v in water) was then visualized and gelatinase activity was quantified by densitometric analysis of the clear bands using software NIH Image J 1.43U (Wayne Rasband, Bethesda, MD) (Frankowski et al., 2012).

#### *In vitro* skin permeation and deposition study

Vertical diffusion cells with effective diffusional area of 1.13 cm^2^ and 3.5 mL of receptor compartment volume were used to perform *in vitro* skin permeation studies. On the previous day of the diffusion study, male Wistar rats (body weight: 180–220 g) were anesthetized by ketamine and the abdominal skin shaved with electric clippers. On the next day, the skin from shaved part was excised and extra tissues removed. The skin was clamped between the donor and receptor compartments with stratum corneum facing the donor compartment. One mg EGCG, in plain solution or transfersomal formulation (EGCG TF, ETF20) was taken in the donor compartment. The receptor compartment was filled with 3.5 mL of phosphate buffer pH 6.8. Samples (500 μL) were withdrawn from the receptor compartment at predetermined time points (0.5, 1, 2, 4, 6, 8, 10 and 12 h) and an equal volume of buffer was replaced each time into the receptor compartment. EGCG content was measured by HPLC. At the end of 12 h, the skin was taken out from the diffusion cell and gently cleansed using 50% v/v methanol to wash off the drug residue. The skin was wiped dry, scissor-cut into small pieces and homogenized with 5 mL of phosphate buffer pH 6.8. The resultant mixture was passed through a 0.45 μm membrane and the filtrate was analyzed for EGCG using HPLC (Mutalik et al., 2012; Mutalik et al., 2014).

#### Statistical analysis

Statistical analysis was performed using GraphPad Prism software (version 5.0, GraphPad, San Diego, CA). For statistical analysis, comparisons were made by one-way ANOVA (analysis of variance) followed by Dunnett’s post-test. *p *< 0.05 was considered statistically significant.

## Results and discussion

### Optimization of transfersomes

Transfersomes containing EGCG and HA were prepared and optimized by DoE using Box–Behnken experimental design. Independent variables selected in this study were amount of phospholipids (*X*_1_), amount of surfactant (*X*_2_), amount of EGCG (*X*_3_) and amount of HA (*X*_4_); whereas dependent variables were vesicle size (*Y*_1_), polydispersity index (*Y*_2_), zeta potential (*Y*_3_), % entrapment efficiency (EE) of EGCG (*Y*_4_) and % EE of HA (*Y*_5_). Dependent variables are important key parameters to optimize the formulations, as shown in Table 1.

#### Effect of drug concentration

In the present study, % EE was affected by the total amount of EGCG and HA incorporated into the transfersomes. EGCG was dissolved along with lipid and surfactant mixture, while HA was dissolved in the hydration medium. The maximum and minimum % EE values for EGCG were found to be 76.53 ± 2.68 (ETF20) and 42.21 ± 2.34 (ETF26), respectively (Table 1). When EGCG concentration was increased from 5 mg (ETF17) to 10 mg (ETF20) per batch, the % EE also increased considerably. But further increase in EGCG amount to 15 mg (ETF2) resulted in a decreased % EE, which may be due to the leakage of EGCG from the vesicle (Ahad et al., 2012). HA was loaded in a quantity ranging from 0.5 mg to 3 mg. The % EE of HA was found to be highest and lowest with ETF9 (57.00 ± 4.98%) and ETF26 (9.00 ± 1.18%), respectively. The highest % EE for HA was found at the amount of 0.5 mg. When the loading concentration of HA was increased from 0.5 mg (ETF5) to 1.75 mg (ETF20), the % EE of HA increased considerably. But further increase to 3 mg (ETF17) resulted in decreased % EE with lipid and surfactant ratio kept constant. This may be due to loss of HA through leakage from the vesicle.

Similarly, the amount of EGCG also influenced the mean particle size and zeta potential of vesicles. The maximum and minimum mean particle size of vesicles was found to be 234.6 ± 8.71 nm (ETF13) and 91.5 ± 6.12 nm (ETF24), respectively (Table 1). When EGCG concentration was increased from 5 mg (ETF17) to 10 mg (ETF20), the mean particle size of the vesicles decreased from 132.8 ± 5.03 nm to 101.2 ± 4.82 nm. But further increase in EGCG concentration to 15 mg (ETF2) resulted in increased mean vesicular size of 176.7 ± 8.20 nm. When HA concentration was increased from 0.5 mg (ETF5) to 1.75 mg (ETF20), the mean size of the vesicles was not decreased substantially (103.7 ± 7.53 nm to 101.2 ± 4.82). But further increased HA concentration to 3 mg (ETF17) resulted in increased mean vesicular size of 132.8 ± 5.03 nm.

The zeta potential of all transfersomal formulations was in negative range (−30.6 ± 3.69 mV to −60.7 ± 3.04 mV). When EGCG concentration was increased from 5 mg (ETF17) to 10 mg (ETF20), the zeta potential of the vesicles was decreased, although not considerably, from −48.9 ± 4.26 mV to −44.8 ± 4.24 mV. Further increase in EGCG concentration to 15 mg (ETF2) resulted in increased zeta potential of the vesicles (−52.9 ± 4.14 mV). There was not much effect of EGCG loading on zeta potential. These results are in accordance with other published data (Gibis et al., 2012; Rashidinejad et al., 2014). In a similar way, when loading amount of HA was increased from 0.5 mg (ETF5) to 1.75 mg (ETF20), the zeta potential of the vesicles was slightly decreased, from −52.0 ± 3.87 mV to −44.8 ± 4.24 mV; but further increase in loading concentration of HA to 3 mg (ETF17) resulted in increased zeta potential of the vesicles (−48.9 ± 4.26 mV). Incorporation of HA at different concentrations did not affect the zeta potential values to a great extent. Large negative or positive zeta potential values induce repulsive interaction between the vesicles and thus avoiding aggregation of vesicles. Accordingly in the present study, the zeta potential of all the formulations was found to be less than −30 mV, which is considered as an acceptable value for physical stability of the vesicles (Caddeo et al., 2008). Amounts of EGCG and HA were optimized to be 10 mg and 1.75 mg respectively for 100 mg of lipid (ETF20) by considering the vesicle size, zeta potential and percentage of EE.

#### Effect of SPC/sodium cholate ratio

Sodium cholate is a widely used edge activator in the preparation of transfersomes. Initially, the % EE of EGCG and HA was increased considerably with an increase in surfactant concentration from 5 mg (ETF25; 56.89 ± 2.80% for EGCG, 26.00 ± 1.50% for HA) to 15 mg (ETF20; 76.53 ± 2.68% for EGCG, 48.57 ± 4.35% for HA) at a constant amount of drug and SPC. With further increase in surfactant concentration to 25 mg (ETF4), the % EE of both EGCG and HA was decreased considerably (49.36 ± 2.60% for EGCG and 21.85 ± 2.14% for HA), which could be due to the pore formation on vesicle surface leading to loss of drug from the vesicles (Ahad et al., 2012). The transfersome batch (ETF20) prepared with 85 mg of SPC and 15 mg of SC showed maximum % EE for EGCG and HA, i.e. 76.53 ± 2.68% and 48.57 ± 4.35%, respectively. This batch also exhibited low particle size (101.2 ± 4.82 nm); therefore, ETF20 was considered as an optimized batch. Patel et al. (2009) also reported similar kind of effect of phospholipids and edge activator on the EE of curcumin in transfersomal formulation. In that study, the % EE of curcumin increased with increasing SPC concentration and decreased with increasing surfactant concentration within certain limits for the ratio of SPC and surfactant (Patel et al., 2009). We did not increase the quantity of surfactant any further, as it may disrupt the vesicles, resulting in decreased % EE.

Phospholipon 90G (SPC), a commonly used lipid in the formulation of transfersome for transdermal delivery, carries negative charge (Duangjit et al., 2011). In the present study, SPC was used from at quantities of 75–95 mg per batch. When SPC concentration was increased from 75 mg (ETF26) to 85 mg (ETF20), the mean particle size of the vesicles decreased from 135.8 ± 6.74 nm to 101.2 ± 4.82 nm. Further increase in SPC concentration to 95 mg (ETF8) resulted in increased mean particle size of the vesicles (122.7 ± 7.16 nm). The zeta potential of the vesicles with different concentrations of SPC such as 75 mg (ETF26), 85 mg (ETF20) and 95 mg (ETF8) was found to be −47.1 ± 4.85 mV, −44.8 ± 4.24 mV and −45.7 ± 4.59 mV, respectively. The results clearly indicated no noteworthy effect of the amount SPC on zeta potential of vesicles.

#### Fitting of data to the model

Response surface plots and corresponding contour plots which show effects of independent variables such as amount of phospholipids (*X*_1_), amount of surfactant (*X*_2_), amount of EGCG (*X*_3_) and amount of HA (*X*_4_) on dependent variables such as vesicle size (*Y*_1_), polydispersity index(*Y*_2_), zeta potential (*Y*_3_), %EE of EGCG (*Y*_4_) and %EE of HA (*Y*_5_) were analyzed. The optimized formulation of transfersomes containing EGCG and HA (ETF20) was explained quantitatively by applying point prediction method using Design Expert software (Version 9.0.3.1; Stat Ease, Minneapolis, MN).

From the results of the optimization process, it was observed that all five independent variables were found to be fitting in quadratic model. Regression equations (4)–(8) of the quadratic models are given below:

(4) $$Y_{1}=+110.029-1.93X_{1}+40.824X_{3}+4.97D\;+20.19X_{1}X_{3}+29.16X_{3}^{2}$$

(5) $$Y_{2}=+0.499+0.006X_{1}-0.031X_{2}-0.016X_{3}\;-0.0071X_{4}+0.0185X_{1}X_{2}+0.08X_{1}X_{3}-0.014X_{1}X_{4}\;+0.013X_{2}X_{3}+0.066X_{2}X_{4}-0.017X_{3}X_{4}\mathrm{CD}$$

(6) $$Y_{3}=-45.86+3.24X_{1}-1.64X_{2}+0.15X_{3}-0.73X_{4}\;+1.4X_{1}X_{2}+2.9X_{1}X_{3}-1.83X_{1}X_{4}-7.77X_{2}X_{3}\;+2.06X_{2}X_{4}-1.6X_{3}X_{4}$$

(7) $$Y_{4}=+73.005-7.008X_{1}-2.17X_{2}-3.55X_{3}+3.19X_{4}\;-9.31X_{1}X_{2}+1.32X_{1}X_{3}+2.68X_{1}X_{4}+0.81X_{2}X_{3}\;+0.91X_{2}X_{4}+0.608X_{3}X_{4}-8.98X_{1}^{2}\;-10.82X_{2}^{2}-6.57X_{3}^{2}-1.85X_{4}^{2}$$

(8) $$Y_{5}=+4.77-11.72X_{1}+5.103X_{2}-2.17X_{3}+5.48X_{4}\;-6.76X_{1}X_{2}+3.15X_{1}X_{3}+6.95X_{1}X_{4}-7.21X_{2}X_{3}\;-1.26X_{2}X_{4}+0.68X_{3}X_{4}-9.13X_{1}^{2}-10.67X_{2}^{2}\;-8.98X_{3}^{2}-2.15X_{4}^{2}$$

Higher values of standard error for coefficient generally show quadratic nonlinear equation. Usually, a positive value in regression indicates direct relationship to optimization indicating synergetic effect; whereas negative value in regression represents inverse relationship to optimization with antagonistic effect. Concentrations of phospholipid (SPC) and edge activator (SC) showed positive effects on responses such as vesicle size (*Y*_1_), polydispersity index (*Y*_2_) and % EE of EGCG (*Y*_4_) whereas negative effects on zeta potential (*Y*_3_) and % EE of HA (*Y*_5_). The difference between the observed and predicted values (error) was analyzed by ANOVA. The observed error in the data of dependent variables was found within the acceptable range with *p* < 0.05.

### Characterization of transfersomal formulations

#### Vesicle size, size distribution and morphology

The prepared transfersomes ranged from 91.7 ± 5.13 nm to 234.60 ± 8.71 nm in average size and from −30.6 ± 3.69 mV to −60.7 ± 3.04 mV for zeta potential values (Table 1). The polydispersity index was found to range from 0.245 ± 0.06 to 0.48 ± 0.11. The transfersomal formulation (ETF20) with particle size of 101.2 ± 4.82 nm, PDI of 0.245 ± 0.06 and zeta potential of −44.8 ± 4.24 mV was deemed optimized.

**Figure 1.**
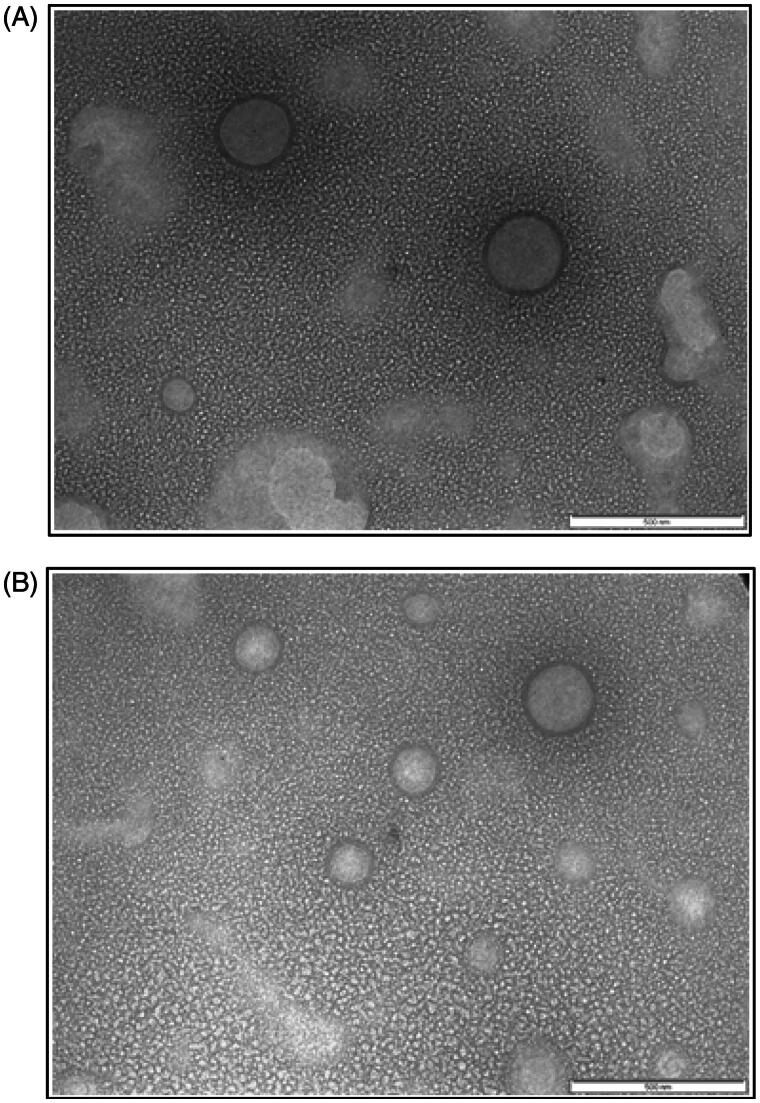
TEM image of (A) blank transfersomes and (B) ETF20 transfersomes (scale: 500 nm).

As discussed in section “Optimization of transfersomes”, the physicochemical properties of the transfersomes were varied with drug/lipid ratios and homogenization pressure, while the volume of hydration medium was kept constant during the preparation of transfersomes. Transmission Electron Microscopic image (Figure 1) of ETF20 showed that the vesicles were spherical in shape with ≈100–200 nm range. In the TEM image, lipidic bilayers of the transfersomes were also distinctively visible without fusion or aggregation. The blank transfersomes (without bioactive agents) also showed similar features (Figure 1).

#### Entrapment efficiency (EE)

EE (%) is also one of the important dependent variables to be optimized in transfersomal formulations. As discussed in section “Optimization of transfersomes”, this parameter strongly depends on the amount of lipid and surfactant, volume of hydration medium and homogenization pressure. The values of % EE of EGCG and HA are given in Table 1. The % EE of EGCG was found to be in the range of 42.21 ± 2.34% (ETF26) to 76.53 ± 2.68% (ETF20). The % EE of HA was found in the range of 9.00 ± 1.18% (ETF26) and 57.00 ± 4.98 (ETF9). High % EE of EGCG is also due to its high lipophilicity, which may strongly lodge it strongly on the surface of the phospholipid bilayers (Ahad et al., 2012).

#### Drug excipient compatibility studies

*FTIR spectroscopy*. EGCG showed characteristic peaks at 3356.25 cm^−1^ and broad stretching between 3570 and 3200 cm^−1^ for O–H group (alcohols). The O–H stretching at 3556.85 cm^−1^ confirms the presence of phenol group. Other characteristic peaks at 1693.56 cm^−1^ (stretching; for C=O group), 1452.45 cm^−1^ (stretching; for aromatic ring), 1344.43 cm^−1^ (for carboxylate stretching), 1227.73 cm^−1^ (stretching; for phenolic C=O), 1147.68–956.72 cm^−1^ (for C–H bending vibrations of aromatic compounds), 823.63–734.9 cm^−1^ (for C–H bending vibrations) and 624.96 cm^−1^ (for alcoholic O–H group) were also observed for plain EGCG.

The spectrum of plain HA showed a broad stretching peak at 3439.19 cm^−1^ representing H bonded OH group, methane C–H stretch at 2899.11 cm^−1^, medium stretching at 1633.76 cm^−1^ for C=O group, a peak at 1413.87 cm^−1^ for carboxylate group and medium stretching peak at 1041.6 cm^−1^ for primary alcohol groups. The representative band at 2920 cm^−1^ corresponding to methylene group was also observed.

The FTIR spectra of the physical mixture of EGCG and HA along excipients and that of the optimized formulation in lyophilized form (ETF20) revealed the characteristic bands of EGCG and HA. These observations indicated the absence of any chemical interactions between the excipients and EGCG and HA in physical blend as well as in the optimized transfersome formulation (Duangjit et al., 2011; Wang & Xia, 2014).

**Figure 2.**
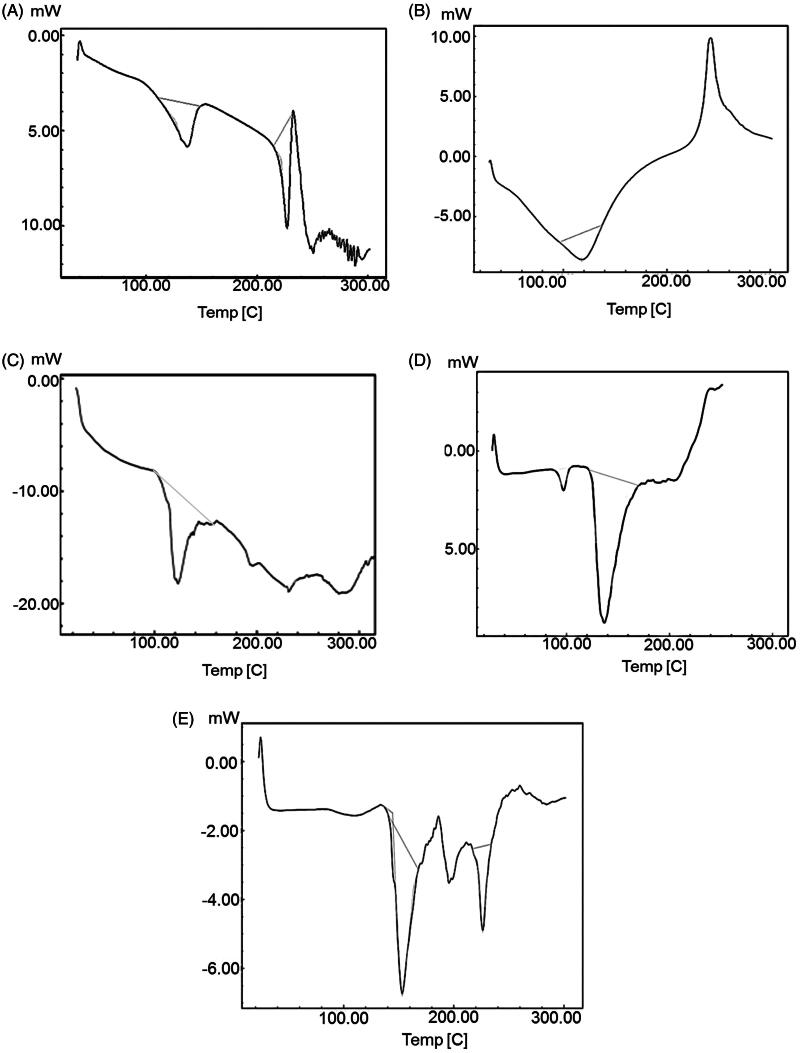
DSC thermograms of (A) EGCG, (B) HA, (C) physical mixture of EGCG and excipients, (D) physical mixture of HA and excipients and (E) optimized transfersome formulation (ETF20).

*Differential scanning calorimetry*. DSC is one of the important techniques to explain physicochemical interaction between the bioactive molecule and excipients of the formulation. Thermodynamic data from DSC is shown in Figure 2. The endothermic peaks of plain EGCG and HA were found at 227.13 °C (Δ*H*: −50.40 J/g) and −118.02 °C (Δ*H*: −80.44 J/g), respectively, corresponding to their melting points as shown in Figure 2(A,B) (Duangjit et al., 2013). The sharp endothermic peak shows crystalline nature and anhydrous state of EGCG and HA. The DSC thermogram of EGCG and its physical mixture with excipients revealed an endothermic peak at 230.8 °C (Δ*H*: −3.37 J/g) as shown in Figure 2(C). The peak intensity was reduced, broadened and slightly shifted which may be due to solubilization of EGCG in the lipid carrier. The DSC thermogram of HA and lipid physical mixture revealed an endothermic peak at 97.15 °C (Δ*H*: −4.11 J/g) as shown in Figure 2(D). There was broadening of the peak with reduction in peak intensity and slight shifting in melting point, which may be again, be due to solubilization of HA in the lipid carrier. Formulation ETF20 also depicted broadened peak at 226.21 °C (Δ*H*: −12.67 J/g) with reduced intensity and absence of HA peak (Figure 2E), which indicates decreased crystalline nature of both EGCG and HA and increased drug salvation in the nano-carrier. The melting point of either EGCG or HA was not considerably altered although there was change in the intensity the peaks. In thermogram of ETF20 transfersomes, enthalpy of heat was also considerably reduced when compared to plain EGCG and HA. Hence, the results of DSC analysis suggest the partial amorphization of both bioactive molecules in the nano-carrier system (Duangjit et al., 2011; Shetty et al., 2015).

**Figure 3.**
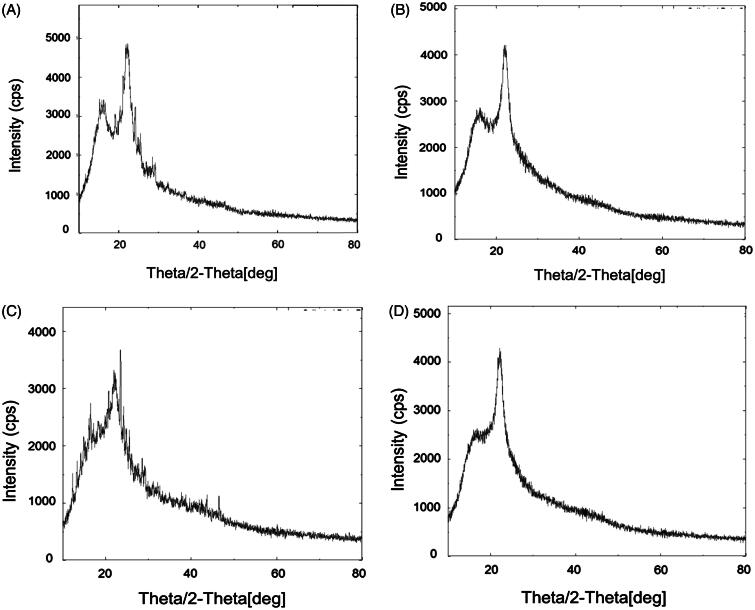
XRD pattern of (A) plain EGCG, (B) plain HA, (C) physical mixture of EGCG, HA and excipients and (D) optimized transfersome formulation (ETF20).

*X-ray diffractometry*. The results of XRD analysis are shown in Figure 3. The XRD pattern of plain EGCG (Figure 3A) revealed the presence of strong diffraction peaks 16.15° (435.12 cps), 21.73° (926.77 cps) and 22.29° (1298.29 cps). The X-ray diffractogram of plain HA (Figure 3B) showed the diffraction peaks at 22.29° (4300.45 cps) and small sharp peaks at 15° to 20°. X-ray diffractogram of physical mixture showed the typical peaks of EGCG with lower intensities because of dominating effect of lipids which are used in the formulations (Figure 3C). Most of the sharp peaks were disappeared and a halo region was observed in XRD pattern of transfersomes (ETF20) containing EGCG and HA (Figure 3D). One peak was remained at 22.2° but was observed with low intensity. XRD analysis confirmed almost complete amorphous nature of EGCG in the transfersome formulation.

### *In vitro* antioxidant activity

The *in vitro* free radical scavenging activity of plain EGCG, EGCG transfersomes (EGCG TF), optimized transfersomes (ETF20) and curcumin (standard) was determined by DPPH and ABTS assays. Plain EGCG and optimized transfersomes showed better IC_50_ values for scavenging both DPPH and ABTS radicals in comparison with curcumin. In this study, the respective IC_50_ values of EGCG for scavenging DPPH and ABTS were found to be 8.52 ± 1.24 μg/mL and 4.12 ± 0.89 μg/mL, which are not noticeably different from those reported previously (Abbas & Wink, 2009). Similarly the IC_50_ values of curcumin observed with DPPH and ABTS (19.19 ± 2.13 and 15.03 ± 2.67 μg/mL, respectively) were also found to be closely comparable to those reported in a previous study (Misra et al., 2016). Transfersomal EGCG showed slightly higher, but not significantly different, IC_50_ values in comparison with plain EGCG. While the IC_50_ values with EGCG-TF were 9.08 ± 1.08 and 4.65 ± 0.65 μg/mL for DPPH and ABTS, the same with ETF20 were 9.17 ± 1.78 and 4.44 ± 1.53 μg/mL. This may be due to slow release of EGCG from transfersomal matrix. Almost similar free radical scavenging activity of EGCG-TF, ETF20 and plain EGCG indicates the retention of complete functional architecture of EGCG after its nanoencapsulation.

### Cell viability (MTT) assay

**Figure 4.**
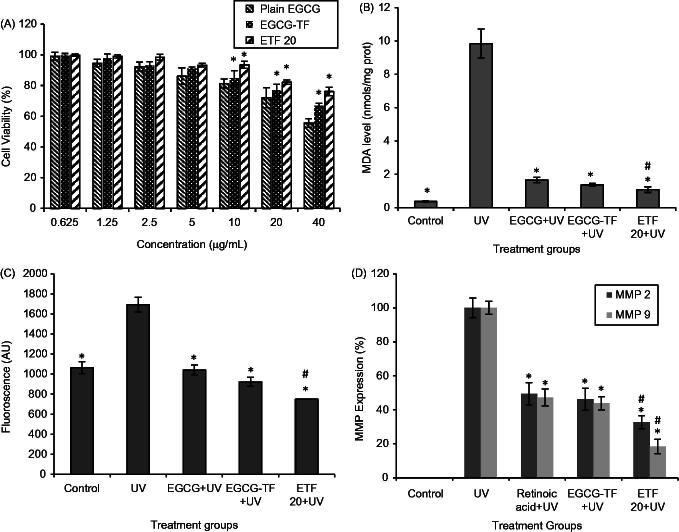
Results of cell viability, MDA, intracellular ROS and MMP expressions. (A) Cell viability (%): *p < 0.05 compared to plain EGCG at that concentration. (B) MDA (nmol, normalized to total cell protein): *p <0.05 compared to UV irradiated group; #*p *< 0.05 compared to plain EGCG + UV. (C) Intracellular ROS (fluorescence values): *p < 0.05 compared to UV irradiation, #*p* < 0.05 compared to all other groups. (D) MMP expressions (%) in HaCaT cells: *p < 0.05 compared to UV irradiated group, #*p *< 0.05 compared to all other groups. All the values are presented as mean ± SD (*n* = 3).

The results of *in vitro* cytotoxicity assay are shown in Figure 4(A). Cytotoxicity of plain EGCG and transfersomes (EGCG-TF and ETF20) was evaluated in HaCaT cells at different concentrations of EGCG (0.625–40 μg/mL). Control (plain buffer/blank transfersomes) showed 100% cell viability. On the other hand, EGCG showed lesser cell viability as its concentration was increased. At 0.625 μg/mL, almost 100% cell viability was observed and at 10 μg/mL concentration of plain EGCG, >80% of the cells were viable; whereas at 40 μg/mL, the cell viability was found to be 55.65%. EGCG transfersomes (EGCG TF) and EGCG + HA transfersomes (ETF20) showed significantly lesser cytotoxic effect on HaCaT cells compared to plain EGCG. At the EGCG concentration of 10 μg/mL, 93.48% and 84.91% of HaCaT cells were viable with ETF20 and EGCG-TF transfersomes, respectively. Similarly, with 40 μg/mL of EGCG, 76.15% and 66.71% of HaCaT cells were viable when treated with ETF20 and EGCG-TF transfersomes, respectively. ETF20 formulation showed higher cell viability compared to EGCG-TF formulation. Lower cytotoxic effect of ETF20 formulation may be due to the presence of HA and/or phospholipid matrix (Liu et al., 2013). These results indicate nontoxic nature of transfersome encapsulated EGCG on HaCaT cells compared to plain EGCG, which are in accordance with previous reports where in some cases encapsulated bioactive molecule showed increased cell viability than in its plain form (Shetty et al., 2015).

### Lipid peroxidation assay

Lipid peroxidation is one of the important mechanistic pathways for UV radiation induced skin cellular injury and it is an indicator of oxidative stress due to photocarcinogenesis and photoaging. Lipid peroxidation is regulated by antioxidant defenses such as polyphenols, GSH, β-carotene, polyunsaturated fatty acids, etc. Elevated levels of lipid peroxidation result from UV radiation, which would result in inactivation of membrane enzymes, loss of fluidity, rupture of cell membrane and DNA damage in skin cells (Hong et al., 2012). Therefore, we used lipid peroxides as markers of photo-oxidative damage. In this study, MDA level in HaCaT cells without UV irradiation (control group) was found to be 0.37 ± 0.05 nmol/mg protein. HaCaT cells irradiated with UV radiation showed significantly (*p* < 0.05) higher MDA level (9.84 ± 0.87 nmol/mg protein). There was significantly decreased level of MDA in the HaCaT cells treated with plain EGCG and the transfersomal formulations (EGCG-TF and ETF20) than that observed in the UV irradiation group (*p *<* *0.05). The values of MDA levels were found to be 1.66 ± 0.17 and 1.37 ± 0.09 nmol/mg protein respectively for plain EGCG and EGCG-TF at the concentration of 10 μg/mL; while the lowest MDA level was found with ETF20 (1.08 ± 0.17 nmol/mg protein) and was significantly (*p *<* *0.05) less than that observed with plain EGCG (Figure 4B). These results clearly indicate that optimized formulation (ETF20) showed more defense mechanism in HaCaT cells against UV irradiation than plain EGCG and EGCG-TF transfersomes. The present results of lipid peroxidation are in accordance with a previous report in which quercetin deformable liposomes showed better improvement in intracellular MDA level in HaCaT cells than plain quercetin (Liu et al., 2013).

### Intracellular ROS level was reduced with optimized formulations

Reactive oxygen species are reactive metabolites capable of being induced by both UV-A and UV-B radiation. ROS are a part of normal cellular regulation and are controlled by cellular antioxidants. However, increase in ROS and decrease in the cellular redox homeostasis can promote carcinogenesis and photoaging, through a variety of mechanisms, including interference with signal cascade systems like the extracellular signal regulated kinase (ERK), p38 kinase, nuclear transcription factor kappa B (NFkB), activated protein-1 (AP-1), phospholipase A2, mitogen-activated protein kinases (MAPKs) and c-Jun N-terminal kinase (JNK). They also modulate stress genes which are associated with cell growth, senescence and transformation of cells to malignant type. Moreover, ROS also upregulate MMPs, accelerating the downregulation of procollagen synthesis (Matés & Sánchez-Jiménez, 2000; Kondo et al., 2004). EGCG has been reported as an effective scavenger of ROS.

In the present study, we compared the protective effect of plain and transfersomal EGCG on UV-induced ROS activity in HaCaT cells, using H_2_DCFDA (Guo et al., 2010; Choi et al., 2015; Farrukh et al., 2015). The results demonstrated that UV irradiated HaCaT cells showed significant (*p* < 0.05) increase in ROS generation which was measured in terms of increased fluorescence intensity as compared to untreated cells (control). This ROS generation was significantly reduced in the cells treated with both plain EGCG and the transfersomes (EGCG TF and ETF20) than UV irradiated cells (*p* < 0.05). In both instances, the intensity of the fluorescence was almost similar to that of control cells (without UV irradiation); however reduction in ROS was more pronounced with ETF20 transfersomal formulation, in which the levels of ROS were significantly (*p* < 0.05) less compared to both plain EGCG as well as EGCG transfersomes (Figure 4C). The better effect of transfersomal formulations in reducing the ROS levels than plain EGCG observed in this study is in agreement with previous reports where quercetin deformable liposomes showed more scavenging activity against UV radiations induced ROS generation in HaCaT cells than plain quercetin (Liu et al., 2013).

### Expression of MMP-2 and MMP-9 by zymography on HaCaT cells

UV radiation induced photoaging is characterized by increased wrinkle formation due to the accumulation and degradation of extracellular proteins such as collagen. Degradation of collagen is normally controlled by the activity of matrix metalloproteinases (MMPs) (Steinbrenner et al., 2003; Subramanian et al., 2014). MMP-2 and MMP-9 are responsible for degradation of type IV and type VII collagen specifically, which are the components of epidermal basement membrane. Gelatinase activity increases under UV irradiation and results in cleavage of basement membrane components, including type IV and type VII collagen, causing detachment and disruption of the basement membrane structure (Inomata et al., 2003).

The results of MMP-2 (72-kDa gelatinase A) and MMP-9 (92-kDa gelatinase B) expression by zymography in HaCaT cells are shown in Figure 4(D). In this study, MMP-2 and MMP-9 expression of 49.4 ± 6.67 and 47.2 ± 3.9% for retinoic acid (standard), 46.2 ± 6.5 and 47.2 ± 4.98% for EGCG-TF and 32.7 ± 3.89 and 18.4 ± 4.2% for ETF20, respectively were obtained in comparison with plain UV irradiated cells. Both nano-transfersomes showed better suppression of MMPs than retinoic acid. As MMP inhibitor, EGCG conceals ultra-structural changes of the basement membrane upon topical administration (Demeule et al., 2000). The result of this study clearly indicated decreased degradation of type IV and type VII collagen and hence is an evidence for reduction in UV-induced aging effect brought about by the transfersomes. Among the two transfersomes, ETF20 (containing both EGCG and HA) exhibited highest suppression of expression of MMPs, which was significantly (*p* < 0.05) more than that found with both plain EGCG as well as EGCG transfersomes. HA plays an important role in physiological functions of skin such as tissue hydration and mechanical protection due to its physicochemical properties like strong hydration and viscoelasticity. In a previous report, the progressive loss of HA present in the skin was observed in UV radiation-induced skin aging conditions (Di Cerbo et al., 2015). The external supply of HA from ETF20 transfersomes could be the reason for the better anti-aging effect produced by this formulation, in comparison with EGCG-TF formulation.

### *In vitro* skin permeation and deposition study

**Figure 5.**
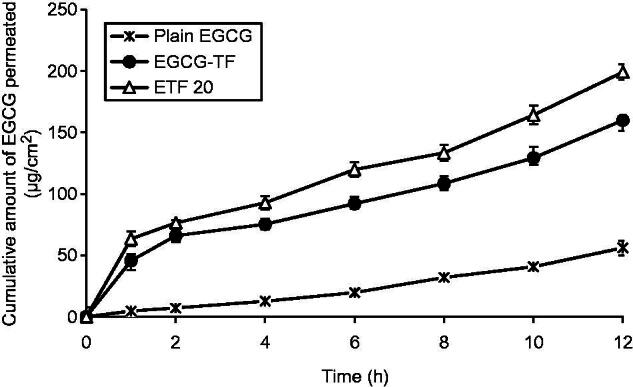
*In vitro* skin permeation profiles of plain EGCG solution, transfersomes containing EGCG and transfersomes containing EGCG and HA (ETF20). Results are presented as mean ± SD (*n* = 3).

The skin permeation profiles of plain EGCG and its transfersomal formulations are shown in Figure 5. EGCG solution exhibited a Q_12_ (cumulative amount of EGCG permeated at the end of 12 h) value of 56.08 ± 4.42 μg/cm^2^; on the other hand EGCG-TF (EGCG alone) transfersomes showed a Q_12_ value of 159.90 ± 6.90 μg/cm^2^, indicating ≈2.8 times enhancement in EGCG permeation. Improvement in skin permeation of drug by using transfersomes as nano-carriers has been well documented. Various drugs such as ketotifen, catechin, valsartan, quercetin, metronidazole and asenapine maleate have shown remarkably higher skin permeation when presented in transfersomal form (Elsayed et al., 2007; Huang et al., 2011; Ahad et al., 2012; Liu et al., 2013; Vanić et al., 2013; Shreya et al., 2015). This improved skin permeation of EGCG from transfersomes may be attributed to flexible structure and small size of these vesicles. Transfersomes penetrate into intercellular lipids and increase the lipid fluidity and decrease the density of lipid layers. The driving force for transfersomes could be the transepidermal water concentration gradient as mentioned in earlier reports. Similar mechanisms might be attributed in the present study also.

An important finding of this study was that co-encapsulation of HA along with EGCG resulted in much higher enhancement of skin permeation of EGCG. Q_12_ value of EGCG from the transfersomes containing EGCG and HA (ETF20) was found to be 199.1 ± 6.30 μg/cm^2^. This Q_12_ value is ≈3.5 times and ≈1.25 times higher than Q_12_ values of plain EGCG (from solution) and EGCG-TF transfersomes, respectively. HA has been reported to possess moisturizing effect, which keeps the skin hydrated and thereby enhances the skin permeation of drugs (Polaskova et al., 2015). Similar mechanism could be responsible for the enhanced skin permeation of EGCG when HA was co-entrapped in transfersomes. These results indicated that along with its inherent anti-aging property, HA has a valuable role in enhancing the skin permeation of EGCG. This observation is supported by the favorable results obtained with ETF20 formulation with respect to free radical (ABTS and DPPH) scavenging, lipid peroxidation, intracellular ROS levels and suppression of MMP expression, which are indicative of antioxidant and anti-aging properties.

The amount of EGCG deposited in the skin with plain EGCG (as solution) was found to be 3.938 ± 0.18 μg/cm^2^. Transfersomes containing EGCG alone (EGCG-TF) showed a significantly higher (*p* < 0.05) EGCG deposition in skin (28.49 ± 1.74 μg/cm^2^) than that observed with plain EGCG solution. Similar observation has been reported in previous literature where transfersomes increased the skin deposition of drugs like cyclosporin A, estradiol, dipotassium glycyrrhizinate, dexamethasone and ketotifen along with enhancement in permeation rate in their transfersomal forms due to transcutaneous hydration gradient (Maghraby et al., 1999; Guo et al., 2000; Trotta et al., 2002; Jain et al., 2003; Elsayed et al., 2007). Further enhancement in skin deposition of EGCG (38.90 ± 1.16 μg/cm^2^) was observed with the transfersomal formulation containing both EGCG as well as HA (ETF20). As reported previously, viscoelastic nature of HA might be helpful in increasing the skin deposition of EGCG (Gao et al., 2014).

## Conclusions

Novel nano-transfersomes containing two bioactive molecules, EGCG and HA, were successfully prepared and statistically optimized using the principles of DoE. The optimized transfersomal formulation containing EGCG and HA (ETF20), showed admirable free radical-scavenging effect and negligible cell toxicity. Moreover, this formulation was able to suppress MDA level and ROS level to a significant extent in human keratinocytes. This formulation was found to suppress the expression level of MMP-2 and MMP-9 in HaCaT cells when compared to standard (retinoic acid) and EGCG-TF transfersomes. Also, higher skin permeation and deposition of EGCG were produced by the transfersomes, in comparison with plain EGCG. Interestingly, the co-entrapment of HA in the formulation increased both, the skin permeation and deposition of EGCG. This study demonstrates the usefulness of the transfersomes loaded with EGCG and HA in topical preparations for multiple advantages. These nano-transfersomes are expected to synergize the UV radiation protection property besides providing antioxidant and anti-aging effects and the studies in this direction are under progress.
